# Soluble urokinase receptor (suPAR) predicts microalbuminuria in patients at risk for type 2 diabetes mellitus

**DOI:** 10.1038/srep40627

**Published:** 2017-01-16

**Authors:** Martina Guthoff, Robert Wagner, Elko Randrianarisoa, Erifili Hatziagelaki, Andreas Peter, Hans-Ulrich Häring, Andreas Fritsche, Nils Heyne

**Affiliations:** 1 Dept. of Endocrinology and Diabetology, Angiology, Nephrology and Clinical Chemistry, University of Tübingen, Otfried-Müller-Str. 10, 72076 Tübingen, Germany; 2 Institute for Diabetes Research and Metabolic Diseases of the Helmholtz Center Munich at the University of Tübingen, Otfried-Müller-Str. 47, 72076, Tübingen, Germany; 3 German Center for Diabetes Research (DZD e.V.), Neuherberg, Germany; 4 Dept. of Internal Medicine II, Research Institute and Diabetes Center, Athens University, Attikon University General Hospital, Athens, Greece

## Abstract

Early identification of patients at risk of developing diabetic nephropathy is essential. Elevated serum concentrations of soluble urokinase receptor (suPAR) associate with diabetes mellitus and predict onset and loss of renal function in chronic kidney disease. We hypothesize, that suPAR may be an early risk indicator for diabetic nephropathy, preceding microalbuminuria. The relationship of baseline suPAR and incident microalbuminuria was assessed in a prospective long-term cohort of subjects at increased risk for type 2 diabetes (TULIP, *n* = 258). Association with albuminuria at later stages of disease was studied in a cross-sectional cohort with manifest type 2 diabetes (ICEPHA, *n* = 266). A higher baseline suPAR was associated with an increased risk of new-onset microalbuminuria in subjects at risk for type 2 diabetes (hazard ratio 5.3 (95% CI 1.1–25.2, *p* = 0.03) for the highest vs. lowest suPAR quartile). The proportion of subjects with prediabetes at the end of observation was higher in subjects with new-onset microalbuminuria. suPAR consistently correlated with albuminuria in a separate cohort with manifest type 2 diabetes. Elevated baseline suPAR concentrations independently associate with new-onset microalbuminuria in subjects at increased risk of developing type 2 diabetes. suPAR may hence allow for earlier risk stratification than microalbuminuria.

Type 2 diabetes mellitus and sequelae constitute a major health problem, affecting more than 29 million people in the US and almost 60 million in Europe^1^. Diabetes mellitus is the principal cause of end stage renal disease (ESRD) requiring renal replacement therapy^2^,^3^. It is hence important to identify patients at risk of developing diabetic nephropathy. Microalbuminuria is considered the first detectable sign of renal involvement in diabetes and used for screening of incipient diabetic nephropathy^4^,^5^. However, risk of progression to diabetic nephropathy as well as associated cardiovascular risk is considered a continuum, starting in the range of normal urinary albumin excretion^6^,^7^. More sensitive biomarkers are hence required to detect patients at risk at an earlier time point and improve efficacy of preventive strategies. Whereas in acute kidney injury, several biomarkers have been proposed for early risk stratification, there are no biomarkers identified for chronic kidney disease, especially for early detection of diabetic nephropathy.

Urokinase-type plasminogen activator receptor (uPAR) is a glycosylphosphatidylinositol (GPI)-anchored receptor of urokinase and expressed on immune cells as well as on endothelial cells, fibroblasts and podocytes^8^,^9^. Its soluble form (suPAR) is formed by cleavage of the membrane-bound GPI anchor and is detectable in various extracellular compartments^8^,^10^. suPAR is a three domain protein with a molecular weight of 20–50 kD, depending on its degree of glycosylation^11^. uPAR/suPAR compounds are involved in inflammation and immune response^12^ and elevated serum suPAR concentrations have been described in malignancies and infections^8^.

As a first hallmark in nephrology, suPAR was described a circulating factor in focal segmental glomerulosclerosis (FSGS) in 2011^11^. Recently, Hayek and coworkers first described a predictive role of plasma suPAR for the development of chronic kidney disease (CKD) in a risk cohort of patients with cardiovascular disease^13^. This finding was especially pronounced in patients with normal renal function at baseline, strengthening the value of suPAR as an early risk indicator for the development of CKD.

As a marker of inflammation, suPAR has also been investigated in the context of metabolic syndrome and diabetes. Higher suPAR concentrations at baseline have been associated with incident type 2 diabetes in prospective analyses^14^,^15^. In manifest diabetes, suPAR was associated with diabetes-related complications in a cross-sectional analysis^16^.

The link between suPAR, albuminuria and diabetes together with the finding of a predictive value for CKD intrigued us to assess the potential of suPAR in predicting onset of albumin excretion in patients at risk for type 2 diabetes. We hypothesized that suPAR is an earlier marker of incipient diabetic nephropathy than microalbuminuria.

## Results

We investigated two cohorts from the Tübingen University Hospital. The TULIP prospective cohort with subjects at increased risk of developing type 2 diabetes (*n* = 258) to address the question whether suPAR concentrations predict onset of microalbumiuria and the ICEPHA cross-sectional cohort, consisting of patients with manifest type 2 diabetes (*n* = 266), to confirm an association of suPAR and urinary albumin excretion at later stages of the disease. Respective baseline characteristics are given in Table 1.

### Distribution of serum suPAR concentrations

**Figure 1:**
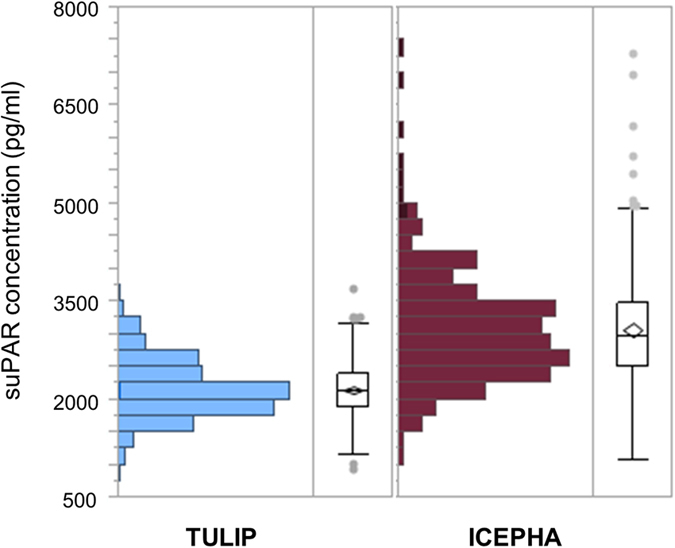
Distribution of suPAR concentrations in cohorts. Distribution of suPAR concentrations in respective cohorts (TULIP prospective cohort and ICEPHA cross-sectional cohort).

In the TULIP cohort, baseline suPAR concentrations ranged from 946 pg/ml to 3701 pg/ml with a median of 2112 [1871–2383] pg/ml. In the ICEPHA cohort with manifest type 2 diabetes, suPAR concentrations were significantly higher with a median of 2974 [2497–3482] pg/ml (*p* < 0.0001). Minimum concentration was 1080 pg/ml, maximum concentration 7306 pg/ml. Distribution of suPAR concentrations is shown in Fig. 1.

### Prospective analysis of suPAR concentrations and new-onset albuminuria

**Figure 2:**
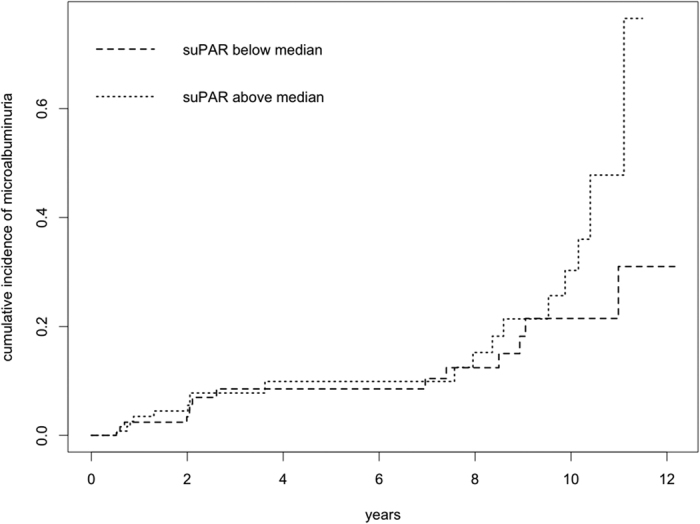
Cumulative hazard of incident microalbuminuria. Cumulative hazard of incident microalbuminuria in longitudinal follow-up of the TULIP prospective cohort.

**Figure 3:**
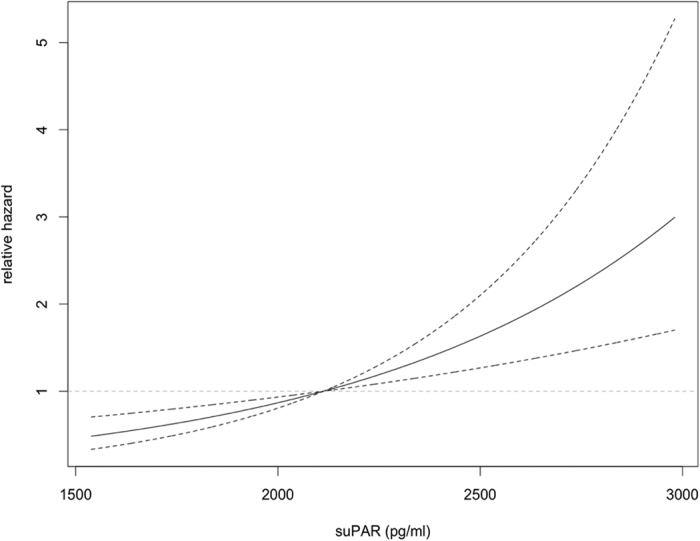
Relative hazard of baseline suPAR for incident microalbuminuria. Non-linear relative hazard function plot (effect estimates from the weighted cox regression model) showing the modeled association between baseline suPAR concentrations and incident microalbuminuria in the TULIP prospective cohort.

In the TULIP cohort, subjects were followed-up for a median of 2.3 [2.0–8.7] years until first onset of microalbuminuria (defined as endpoint) or the last UACR measurement, if no microalbuminuria occured. There were 32 events of new-onset microalbuminuria (12.4%). Initial analysis of the cumulative hazard plot stratified for baseline suPAR below or above the median (2112 pg/ml) showed a separation of the plots after 8 years (Fig. 2). Since the hazard related to baseline suPAR levels tended to increase over time (cf. Supplementary Fig. S1), we performed a weighted Cox regression analysis. In the univariate model, baseline suPAR significantly associated with the incidence of new-onset microalbuminuria (e^ß^ = 1.0011 per pg/ml higher suPAR, *p* = 0.001). A significant interaction between time and baseline suPAR (e^ß^ = 1.0002 per year, *p* = 0.03) suggested that the effect increased over time. After adjustment for baseline eGFR, suPAR remained a significant predictor of new-onset microalbuminuria (*p* = 0.0003). This association was not abolished after further adjustment for baseline blood pressure (expressed as mean arterial pressure, MAP), glycemia (baseline HbA1c) and the use of angiotensin converting-enzyme inhibitors or angiotensin AT_1_-receptor antagonists (ARB) (for suPAR, *p* = 0.0002 in the model with all covariates). The estimated relative hazard for a given baseline suPAR level after adjustment for baseline eGFR, blood pressure, glycemia and nephroprotective medication is displayed in Fig. 3. When comparing quartiles of suPAR, participants in the highest vs. lowest quartile of suPAR had a hazard ratio of 5.3 (CI: 1.1–25.2, *p* = 0.03) for new-onset microalbuminuria.

### Characterization of subjects reaching the endpoint

Characteristics of subjects with new-onset microalbuminuria and those not reaching the endpoint during follow-up are given in Table 2. In subjects reaching the endpoint, median time to incident microalbuminuria was 3.1 years, ranging from 1.5 to 8.8 years. The proportion of females was significantly higher among subjects developing microalbuminuria (*p* = 0.04). Otherwise, there were no significant differences in anthropometrics. At end of follow-up, the number of subjects with prediabetes was higher in those developing microalbuminuria, compared to those who did not. In accordance, HbA1c was significantly higher in these subjects (*p* = 0.03). Median eGFR was comparable among subjects with and without new-onset microalbuminuria. Mean arterial blood pressure or the use of ACEI or ARB did not differ between groups.

### Correlation of serum suPAR concentrations and albuminuria in manifest type 2 diabetes

In an independent cross-sectional cohort of patients with manifest type 2 diabetes (ICEPHA), suPAR concentrations significantly correlated with albuminuria (r^2^ = 0.08, *p* < 0.0001). Higher suPAR levels were associated with an increase in albuminuria.

## Discussion

The main finding of the present investigation is that serum suPAR independently associates with new-onset microalbuminuria in subjects at increased risk for type 2 diabetes. This finding was persistent after adjustment for known risk factors for urinary albumin excretion. In an independent cohort with manifest type 2 diabetes, the association of suPAR and urinary albumin excretion was conserved at later stages of disease.

Microalbuminuria is considered the earliest detectable marker of diabetic nephropathy and state-of-the-art in screening for renal involvement in diabetes mellitus. Microalbuminuria is an independent risk factor for progression to proteinuria^17^ and subsequent loss of renal function^4^. Moreover, the reduction of albumin excretion is a therapeutic target in diabetic nephropathy^18^. Current opinion considers the risk of developing diabetic nephropathy a continuum, starting at urinary albumin excretion still within the normal range^6^,^7^. Detection of incipient diabetic nephropathy at earlier time points is hence essential.

We now propose suPAR a candidate biomarker for this task. This concept is supported by an established pathophysiologic link between suPAR and podocyte integrity. suPAR binds to and activates β_3_-integrin, resulting in podocyte effacement and alteration of glomerular permselectivity^11^. Most data available is from patients with FSGS. In these patients, an association of suPAR and urinary albumin excretion has been shown and extracorporeal elimination of suPAR by plasmapheresis resulted in reduced β_3_-integrin activity and reduction of proteinuria^19^. In animal models of diabetic nephropathy, blockade of β_3_-integrin using monoclonal antibodies inhibits progression of albuminuria^20^. The effect of suPAR on podocyte function may be modulated by other pathways in a disease-specific manner. Unlike in FSGS, expression of sphingomyelinase-like phosphodiesterase 3b (SMPDL3b) is high in diabetic nephropathy, shifting suPAR mediated podocyte injury from a migratory (FSGS) to an apoptotic phenotype (diabetic nephropathy)^21^. Both will result in albuminuria. Clinically, an association of suPAR and urinary albumin excretion in diabetes has been shown in two cross-sectional analyses^16^,^22^. suPAR may hence mediate podocyte dysfunction and the onset of microalbuminuria in diabetic nephropathy.

A first indicator of a predictive role for suPAR in CKD is derived from the work of Hayek and coworkers who recently demonstrated a longitudinal association of baseline suPAR levels with a decline in eGFR and incident CKD in a cohort of patients with cardiovascular disease^13^. We now confirm a predictive role of suPAR in kidney disease in a different clinical setting in patients at increased risk of type 2 diabetes. In this cohort, suPAR predicted the onset of microalbuminuria, as the first clinical sign of renal involvement and clearly upstream of CKD and a decline in GFR. Importantly, this would allow the identification of patients at risk for diabetic nephropathy and CKD years in advance.

In defining the role of suPAR in kidney disease, discerning interactions between suPAR, albuminuria and decline in kidney function is key to gain insight into underlying pathomechanisms and to answer the question, whether suPAR merely associates with renal involvement in longitudinal observations (biomarker) or by itself is a driving force for a decline in renal function (progression factor). Our data now confirm the value of suPAR as a biomarker of kidney disease. Whether suPAR-mediated podocyte injury and albuminuria constitute a potential pathophysiologic link to CKD remains to be addressed in further studies.

As a biomarker, suPAR concentrations are affected by kidney function. With a molecular weight of 20–50 kD, suPAR is subject to glomerular filtration, and a number of investigations demonstrate an inverse relationship between suPAR and eGFR^22^,^23^,^24^. In our data, the association of suPAR with incident microalbuminuria was independent from baseline eGFR.

Only one of the subjects with new-onset microalbuminuria progressed to manifest diabetes throughout follow-up. So are we in fact looking at incipient diabetic nephropathy in our cohort? Renal involvement in diabetes has been shown prior to manifest disease in patients with prediabetes^25^ and in normoglycemic individuals with insulin resistance^26^. Furthermore, triggering factors other than glycemia have been discussed for end-organ manifestations in diabetes^27^,^28^. In our carefully phenoptyped cohort, glycated hemoglobin levels were significantly higher and more subjects showed prediabetes among those developing microalbuminuria than in subjects not reaching the endpoint. Apart from this, no differences in other factors affecting urinary albumin excretion were observed. Taken together, these findings support the notion, that new-onset microalbuminuria in fact reflects incipient diabetic nephropathy in our prospective cohort.

Some limitations do apply to our data. The size of the cohorts is relatively small, albeit homogenous and, as for the TULIP cohort, one of the largest at-risk cohorts with long-term follow-up in the field of type 2 diabetes. Also, the number of subjects reaching an endpoint is small, in part owing to controlled lifestyle intervention. On the other hand, our conclusions are strengthened by a number of aspects: We investigate two carefully defined cohorts, applying the same methodology including urinalysis at different stages of the disease. By doing so, we demonstrate suPAR to be an independent predictor for the onset of microalbuminuria as a key indicator and progression factor of diabetic nephropathy and to consistently correlate to the magnitude of urinary albumin excretion in manifest disease. This not only underscores the relevance of this finding but also allows to hypothesize a potential pathophysiologic link between suPAR, urinary albumin excretion and progression to CKD in diabetic nephropathy, which has to be addressed in future trials. Importantly, the fact that we investigated an at-risk cohort, in which structured lifestyle intervention was performed to reduce the risk of progression, implies that the impact could even be stronger in an everyday clinical setting. Lastly, variance of baseline suPAR levels in the TULIP cohort is small. This reflects the homogeneity of an at-risk cohort for a disease. Most published data have evaluated suPAR in patients with manifest disease, with variable severity and comorbidity, or at increased risk of developing an associated complication, displaying higher variance. In line, our second cohort with manifest type 2 diabetes showed higher absolute levels and variance of suPAR than TULIP.

In conclusion, we for the first time demonstrate that suPAR associates with the onset of microalbuminuria in patients at risk for type 2 diabetes, preceding known markers of renal involvement by several years. An earlier identification of patients at risk for diabetic nephropathy may allow for more effective prevention and improve patient prognosis.

## Methods

### Study population

Participants were studied from two cohorts at the Tübingen University Hospital. The prospective cohort is the Tübingen Lifestyle Intervention Program (TULIP) study. The study was designed to prospectively assess the effect of lifestyle intervention on metabolic phenotype in subjects at increased risk of type 2 diabetes. TULIP included individuals from 2003–2005 with subjects recruited by newspaper invitation or personal recommendation. Inclusion criteria were a family history of type 2 diabetes, a body mass index (BMI) > 27 kg/m^2^, impaired glucose tolerance or a history of gestational diabetes. The TULIP study was approved by the Institutional Ethics Committee of the Medical Faculty of Tübingen (422/2002) and conducted in accordance with the declaration of Helsinki. Written informed consent was obtained from all subjects prior to inclusion. Lifestyle modification consisted of guided dietary modification (intake of less than 30% of total calories as fat, reduction in saturated fatty acids and increased amount of fibers) as well as increased physical activity (3 h of moderate physical activity per week). Participants had planned follow-up visits at the end of lifestyle intervention at 9 months and after 24 months, and were subsequently invited for long-term follow-up. Visits included an extended oral glucose tolerance test to assess metabolic parameters, as well as routine laboratory and urinalysis.

In the TULIP study, fasting venous blood samples were obtained on each occasion and serum and plasma stored at −80 °C. suPAR was measured at baseline examination prior to lifestyle intervention. Urinary albumin-to-creatinine-ratios (UACR) were determined from freshly voided spot urine samples. Samples were collected before the oral glucose tolerance test on each occasion. Incident microalbuminuria was defined as occurrence of UACR ≥30 mg/g creatinine, given this finding was not present at a prior examination. Samples with dipstick leukocyturia were excluded from the analyses as were participants with C-reactive protein levels over 0.5 mg/dl. All participants included in the analysis had a UCAR below 30 mg/g at baseline.

The cross-sectional cohort is from the ICEPHA study, conducted by the Interfaculty Centre for Pharmacogenomics and Pharma Research (ICEPHA) of Tübingen and Stuttgart. The study was planned to establish individualized therapy for patients with type 2 diabetes and is conducted as a registry to assess clinical phenotypes and laboratory parameters related to type 2 diabetes. The study included from 2011–2012 and recruited via regular visit in our diabetes outpatient clinic, without exclusion criteria. Inclusion criterion was manifest type 2 diabetes. The trial was approved by the Institutional Ethics Committee of the Medical Faculty of Tübingen (343/2010BO2) and conducted in accordance with the declaration of Helsinki. Written informed consent was obtained from all patients prior to inclusion in the trial. suPAR was measured at baseline together with routine laboratory including urinalysis.

In the present analysis, we assessed the association of baseline serum suPAR levels and (i) new-onset microalbuminuria as endpoint in long-term follow-up of the TULIP prospective cohort and (ii) the magnitude of urinary albumin excretion in the ICEPHA cross-sectional cohort.

### Analytical measurements and calculations

Serum suPAR concentrations were measured using the Human uPAR Quantikine ELISA kit (R&D Systems, Wiesbaden, Germany) according to the manufacturers instruction. The minimal detectable concentration of suPAR is 33 pg/ml; the inter-assay variation is given as 5.1%, the intra-assay variation as 4.1%. Urinary albumin concentration was measured nephelometrically (BN ProSpec Nephelometer, Siemens Healthcare Diagnostics, Eschborn, Germany) and creatinine quantified enzymatically (ADVIA 1800 Clinical Chemistry System, Siemens Healthcare Diagnostics, Eschborn, Germany). Plasma glucose was measured on the ADVIA 1800 clinical chemistry analyzer (hexokinase method), HbA1c was measured by high performance liquid chromatography (Tosoh G8 HPLC Analyzer, Tokyo, Japan).

UACR was calculated from urinary albumin and creatinine concentrations. For calculation of eGFR, the CKD-EPI equation was used, which takes into account age and gender^29^.

### Statistical analysis

Variables are given as median and interquartile range. Distribution was tested with histograms and Shapiro-Wilk test. Groups were compared using Wilcoxon test for continuous variables and Fisher’s exact test for categorical variables.

To test the hypothesis if baseline suPAR levels predict new-onset microalbuminuria, we intended to use a proportional hazards (Cox) model. Since the effect of baseline suPAR levels tended to increase over time (cf. Supplementary Fig. S1), the proportionality assumption of the original Cox model was violated. To overcome this limitation, we applied the weighted Cox regression method proposed by Schemper, Wakounig and Heinze^30^. Weighted Cox regression provides unbiased average hazard ratio estimates also in case of non-proportional hazards.

To test the correlation of suPAR with albuminuria in manifest type 2 diabetes, univariate linear regression analysis was used. Non-normally distributed parameters were log transformed to approximate normal distribution prior to analysis.

Results with values of p < 0.05 were considered statistically significant. Analyses were performed using JMP 11.0 (SAS Institute, Cary, NC) and R version 3.2.2 (R foundation for statistical computing, Vienna, Austria).

## Additional Information

**How to cite this article**: Guthoff, M. *et al*. Soluble urokinase receptor (suPAR) predicts microalbuminuria in patients at risk for type 2 diabetes mellitus. *Sci. Rep.* **7**, 40627; doi: 10.1038/srep40627 (2017).

**Publisher's note:** Springer Nature remains neutral with regard to jurisdictional claims in published maps and institutional affiliations.

## Supplementary Material

Supplementary Figure S1

## Figures and Tables

**Table 1:** Baseline characteristics of cohorts.

	TULIP prospective cohort	ICEPHA cross-sectional cohort
*n*	258	266
gender (m/f)	110/148	152/114
age (yrs)	47 [39–54]	64 [56–71]
BMI* (kg/m^2^)	29.0 [26.6–31.9]	33.3 [29.5–37.9]
yrs since dx of diabetes	n.a.	9.5 [5–16]
FPG^†^ (mmol/l)	5.2 [4.8–5.6]	8.6 [7.2–10.6]
HbA1c^‡^ (%)	5.6 [5.3–5.9]	7.6 [6.9–9.2]
plasma creatinine (μmol/l)	88.4 [79.6–88.4]	70.7 [61.9–88.4]
eGFR CKD-EPI^§^ (ml/min/1.73 m^2^)	81 [72–93]	90 [66–99]
UACR^II^ (mg/g Crea)	6 [4–9]	16 [8–61]
MAP^¶^ (mmHg)	90 [83–98]	105 [100–110]
ACEI^#^ or ARB** use (*n*) [y/n/unknown]	14/244/0	200/41/25
serum suPAR concentrations (pg/ml)	2112 [1871–2383]	2974 [2497–3482]

Date given as median [interquartile range]; n.a.: not applicable.

*Body mass index, ^†^fasting plasma glucose, ^‡^glycated hemoglobin A1c, ^§^estimated glomerular filtration rate according to CKD-EPI formula^29^, ^II^urinary albumin-to-creatinine ratio, ^¶^mean arterial blood pressure, ^#^angiotensin converting enzyme inhibitor, **angiotensin AT_1_-receptor antagonist.

**Table 2:** Characteristics of subjects reaching the endpoint of new-onset microalbuminuria or not; TULIP prospective cohort.

	Baseline		*p*	End of follow-up		*p*
	Reaching endpoint	Not reaching endpoint		Reaching endpoint	Not reaching endpoint	
*n*	32	226		32	226	
gender (m/f)	8/24	102/124		8/24	102/124	0.04*
duration of follow-up (yrs.)				3.1 [1.5–8.8]	2.3 [2.0–8.7]	0.82
age (yrs)	50 [42–56]	47 [39–54]	0.26	57 [47–61]	52 [43–60]	0.16
BMI* (kg/m^2^)	28.9 [25.8–32.2]	29.0 [26.7–31.9]	0.95	28.7 [25.1–31.7]	29.0 [26.2–32.2]	0.57
FPG^†^ (mmol/l)	5.4 [5.1–5.7]	5.1 [4.8–5.6]	0.11	5.4 [5.2–5.9]	5.3 [4.9–5.6]	0.12
HbA1c^‡^ (%)	5.6 [5.3–6.1]	5.6 [5.3–5.9]	0.22	5.7 [5.5–6.1]	5.6 [5.3–5.9]	0.03*
prediabetes^§^ (*n*)	17	141	0.34	25	138	0.08
eGFR CKD-EPI^II^ (ml/min/1.73 m^2^)	84 [69–99]	81 [72–92]	0.61	95 [90–102]	94 [82–104]	0.45
UACR^¶^ (mg/g Crea)	<30	<30		39 [34–75]	8 [5–14]	<0.001*
MAP^#^ (mmHg)	97 [86–101]	90 [83–98]	0.08	100 [87–108]	95 [89–103]	0.24
ACEI** or ARB^††^ use (*n*) [y/n]	3/32	11/226	0.39	3/32	11/226	0.39

Date are given as median [interquartile range]. *p* values among groups at baseline or end of follow-up, respectively.

*Body mass index, ^†^fasting plasma glucose, ^‡^glycated hemoglobin A1c, ^§^according to American Diabetes Association (ADA) criteria, ^II^estimated glomerular filtration rate according to CKD-EPI formula^29^, ^¶^urinary albumin-to-creatinine ratio, ^#^mean arterial blood pressure, **angiotensin converting enzyme inhibitor, ^††^angiotensin AT_1_-receptor antagonist.

## References

[b1] International Diabetes Federation. IDF Diabetes Atlas 7th edition. http://www.diabetesatlas.org (Date of access: 18.10.2016) (2015).

[b2] United States Renal Data System. Annual Data Report 2015. https://www.usrds.org/2015/view/Default.aspx (Date of access: 18.10.2016) (2015).

[b3] ERA-EDTA Registry, Annual Report 2014. https://www.era-edta-reg.org/files/annualreports/pdf/AnnRep2014.pdf (Date of access: 18.10.2016) (2016).

[b4] Gross J. L. . Diabetic nephropathy: diagnosis, prevention, and treatment. Diabetes Care 28, 164–176 (2005). 15616252 10.2337/diacare.28.1.164

[b5] Sacks D. B. . Guidelines and recommendations for laboratory analysis in the diagnosis and management of diabetes mellitus. Diabetes Care 34, e61–99 (2011). 21617108 10.2337/dc11-9998 PMC3114322

[b6] Murussi M., Baglio P., Gross J. L. & Silveiro S. P. Risk factors for microalbuminuria and macroalbuminuria in type 2 diabetic patients: a 9-year follow-up study. Diabetes Care 25, 1101–1103 (2002). 12032128 10.2337/diacare.25.6.1101

[b7] Predictors of the development of microalbuminuria in patients with Type 1 diabetes mellitus: a seven-year prospective study. The Microalbuminuria Collaborative Study Group. *Diabet. Med.* **16,** 918–925 (1999). 10588521

[b8] Thunø M., Macho B. & Eugen-Olsen J. suPAR: the molecular crystal ball. Dis. Markers 27, 157–172 (2009). 19893210 10.3233/DMA-2009-0657 PMC3835059

[b9] Wei C. . Modification of kidney barrier function by the urokinase receptor. Nat. Med. 14, 55–63 (2008). 18084301 10.1038/nm1696

[b10] Mizukami I. F., Faulkner N. E., Gyetko M. R., Sitrin R. G. & Todd R. F. Enzyme-linked immunoabsorbent assay detection of a soluble form of urokinase plasminogen activator receptor *in vivo*. Blood 86, 203–211 (1995). 7795225

[b11] Wei C. . Circulating urokinase receptor as a cause of focal segmental glomerulosclerosis. Nat. Med. 17, 952–960 (2011). 21804539 10.1038/nm.2411 PMC4089394

[b12] Montuori N., Visconte V., Rossi G. & Ragno P. Soluble and cleaved forms of the urokinase-receptor: degradation products or active molecules? Thromb. Haemost. 93, 192–198 (2005). 15711732 10.1160/TH04-09-0580

[b13] Hayek S. S. . Soluble Urokinase Receptor and Chronic Kidney Disease. N. Engl. J. Med. 373, 1916–1925 (2015). 26539835 10.1056/NEJMoa1506362 PMC4701036

[b14] Haugaard S. B. . The immune marker soluble urokinase plasminogen activator receptor is associated with new-onset diabetes in non-smoking women and men. Diabet. Med. 29, 479–487 (2012). 22050462 10.1111/j.1464-5491.2011.03513.x

[b15] Heraclides A. . The pro-inflammatory biomarker soluble urokinase plasminogen activator receptor (suPAR) is associated with incident type 2 diabetes among overweight but not obese individuals with impaired glucose regulation: effect modification by smoking and body weight status. Diabetologia 56, 1542–1546 (2013). 23613086 10.1007/s00125-013-2914-0

[b16] Theilade S. . Soluble urokinase plasminogen activator receptor levels are elevated and associated with complications in patients with type 1 diabetes. J. Intern. Med. 277, 362–371 (2015). 24830873 10.1111/joim.12269

[b17] Mogensen C. E. Microalbuminuria predicts clinical proteinuria and early mortality in maturity-onset diabetes. N. Engl. J. Med. 310, 356–360 (1984). 6690964 10.1056/NEJM198402093100605

[b18] Imai E. . Reduction and residual proteinuria are therapeutic targets in type 2 diabetes with overt nephropathy: a post hoc analysis (ORIENT-proteinuria). Nephrol. Dial. Transplant. 28, 2526–2534 (2013). 24013685 10.1093/ndt/gft249

[b19] Staeck O. . Recurrent Primary Focal Segmental Glomerulosclerosis Managed With Intensified Plasma Exchange and Concomitant Monitoring of Soluble Urokinase-Type Plasminogen Activator Receptor-Mediated Podocyte β3-integrin Activation. Transplantation 99, 2593–2597 (2015). 26371597 10.1097/TP.0000000000000914 PMC4900174

[b20] Maile L. A. . Blocking αVβ3 integrin ligand occupancy inhibits the progression of albuminuria in diabetic rats. J. Diabetes Res. 2014, 421827 (2014). 25389530 10.1155/2014/421827 PMC4217341

[b21] Yoo T.-H. . Sphingomyelinase-like phosphodiesterase 3b expression levels determine podocyte injury phenotypes in glomerular disease. J. Am. Soc. Nephrol. 26, 133–147 (2015). 24925721 10.1681/ASN.2013111213 PMC4279736

[b22] Wu C.-Z. . Urokinase plasminogen activator receptor and its soluble form in common biopsy-proven kidney diseases and in staging of diabetic nephropathy. Clin. Biochem. 48, 1324–1329 (2015). 26162494 10.1016/j.clinbiochem.2015.07.001

[b23] Meijers B. . The soluble urokinase receptor is not a clinical marker for focal segmental glomerulosclerosis. Kidney Int. 85, 636–640 (2014). 24402090 10.1038/ki.2013.505

[b24] Wada T. . A multicenter cross-sectional study of circulating soluble urokinase receptor in Japanese patients with glomerular disease. Kidney Int. 85, 641–648 (2014). 24429394 10.1038/ki.2013.544

[b25] Singleton J. R., Smith A. G., Russell J. W. & Feldman E. L. Microvascular complications of impaired glucose tolerance. Diabetes 52, 2867–2873 (2003). 14633845 10.2337/diabetes.52.12.2867

[b26] Mykkänen L. . Microalbuminuria is associated with insulin resistance in nondiabetic subjects: the insulin resistance atherosclerosis study. Diabetes 47, 793–800 (1998). 9588452 10.2337/diabetes.47.5.793

[b27] Hidmark A. . A new paradigm to understand and treat diabetic neuropathy. Exp. Clin. Endocrinol. Diabetes 122, 201–207 (2014). 24623503 10.1055/s-0034-1367023

[b28] Humpert P. M. . Soluble RAGE but not endogenous secretory RAGE is associated with albuminuria in patients with type 2 diabetes. Cardiovasc. Diabetol. 6, 9 (2007). 17343760 10.1186/1475-2840-6-9 PMC1821011

[b29] Levey A. S. . A new equation to estimate glomerular filtration rate. Ann. Intern. Med. 150, 604–612 (2009). 19414839 10.7326/0003-4819-150-9-200905050-00006 PMC2763564

[b30] Schemper M., Wakounig S. & Heinze G. The estimation of average hazard ratios by weighted Cox regression. Stat. Med. 28, 2473–2489 (2009). 19472308 10.1002/sim.3623

