# Supplementary material for: Soluble urokinase receptor (suPAR) predicts microalbuminuria in patients at risk for type 2 diabetes mellitus
Source: Sci Rep. 2017 Jan 16;7:40627. doi: 10.1038/srep40627 (PMC5238426; doi:10.1038/srep40627)

Journal: Scientific Reports

Soluble urokinase receptor (suPAR) predicts microalbuminuria in patients at risk for type 2 diabetes mellitus

Martina Guthoff [†], Robert Wagner [†], Elko Randrianarisoa, Erifili Hatziagelaki, Andreas Peter, Hans-Ulrich Häring, Andreas Fritsche and Nils Heyne

[†] both authors contributed equally to this work

Supplementary figure 1: Assessment of the proportional hazards assumption in a Cox regression setting

Schoenfeld residuals plot indicating a time-dependent effect for suPAR and violation of the proportional hazards assumption

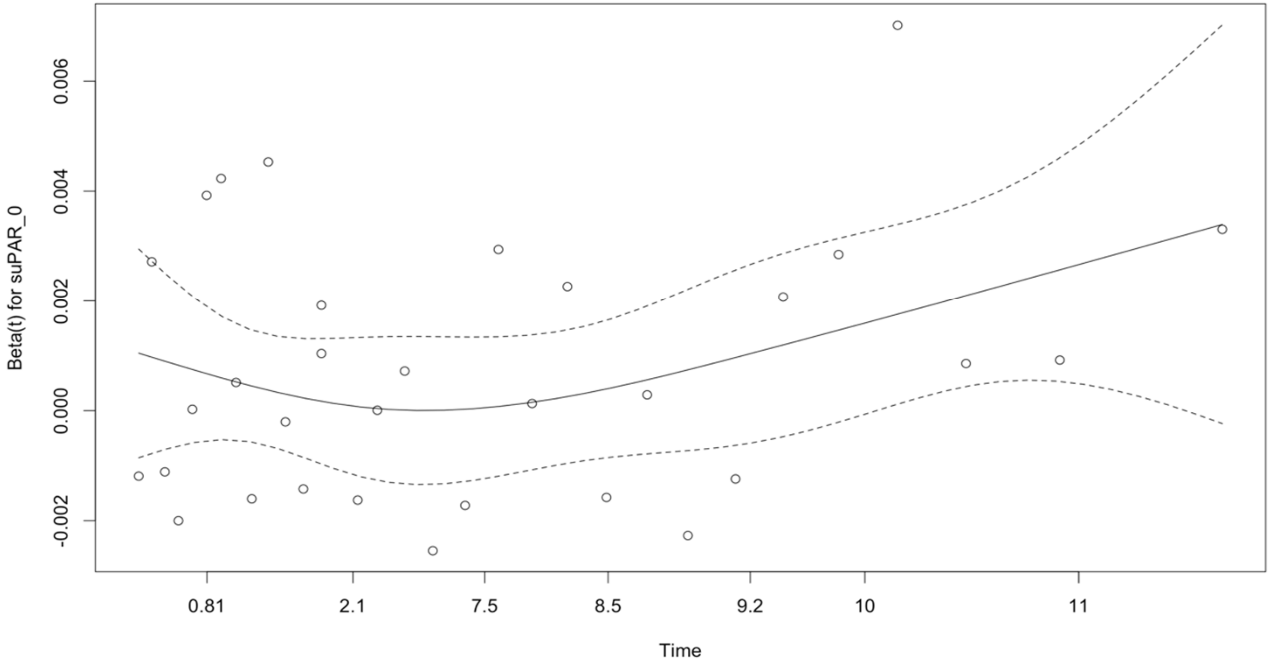

Supplement: Supplementary Figure S1 [file srep40627-s1.pdf]
